# Optimization of Different Extraction Methods for Phenolic Compound Verbascoside from Chinese *Olea europaea* Leaves Using Deep Eutectic Solvents: Impact on Antioxidant and Anticancer Activities

**DOI:** 10.3390/molecules29174219

**Published:** 2024-09-05

**Authors:** Yan Deng, Junlin Zhou, Bixia Wang, Xiao Xu, Tingyu Huang, Zhou Xu, Chunyan Zhao

**Affiliations:** 1 College of Life Science, Environmental Science and Engineering, China West Normal University, Nanchong 637009, China; 18121927870@163.com (Y.D.); 18282097686@163.com (J.Z.); 13684327650@163.com (T.H.); 2 Panxi Crops Research and Utilization Key Laboratory of Sichuan Province, Xichang University, Xichang 615000, China; xzhbiol@163.com; 3 Sichuan Yizhang Agricultural Development Co., Ltd., Nanchong 637009, China; 17883305560@163.com

**Keywords:** *Olea europaea* leaves, verbascoside, ultrasound-assisted extraction, wall-breaking extraction, antioxidant activity, anticancer activity

## Abstract

Chinese *Olea europaea* leaves, rich in verbascosides, were extracted using ultrasound-assisted extraction (UAE) and wall-breaking extraction (WBE) with deep eutectic solvents (Optimal UAE: 55 min, 200 mL/g liquid–solid ratio, 20% moisture, yielding 206.23 ± 0.58 mg GAE/g total phenolic content (TPC) and 1.59 ± 0.04% verbascoside yield (VAY); Optimal WBE: 140 s, 210 mL/g, 30% moisture, giving 210.69 ± 0.97 mg GAE/g TPC and 1.33 ± 0.2% VAY). HPLC analysis showed that young leaves accumulated higher TPC and phenolic compounds. Among the five olive varieties, Koroneiki and Chemlal showed the highest TPC in UAE, while Arbosana and Chemlal excelled in WBE. WBE yielded a higher TPC and rutin, whereas UAE marginally increased other phenolics. Additionally, the DPPH^•^ assay showed that WBE-extracted verbascoside-rich extracts (VREs) of Chemlal exhibited high antioxidant activity (EC_50_ of 57 mg/mL), but Koroneiki-VREs exhibited lower activity against the ABTS^•^**^+^** radical (EC_50_ of 134 mg/mL). Remarkably, the UAE/WBE-extracted Chemlal-VREs promoted the normal esophageal Het-1A cell line at 25 μg/mL for 24 h; yet, the esophageal cancer Eca-109 cells were sensibly inhibited, especially at 50 μg/mL; and the cell viability decreased dramatically. The results confirmed WBE as a relatively efficient method, and the Chemlal variety may be an excellent source of verbascoside.

## 1. Introduction

Phenolic compounds are widely regarded as the primary agents responsible for plants’ therapeutic properties [1]. Verbascoside, a common polyphenolic compound found in plants [2], has been extensively noted for its apparent role in preventing and treating various conditions and diseases. It exhibits antidiabetic [3], antioxidant [4], anticancer [5,6], and anti-inflammatory [7,8] properties. Moreover, verbascoside stands out as a key bioactive compound, aiding in the prevention or treatment of cardiovascular and neurological diseases, kidney and nerve protection, and a reduction in blood sugar and lipids [6,8,9]. Given its vast potential in disease management, sources of verbascoside are continuously being explored. However, studies reveal that natural plants often contain low concentrations of verbascoside, typically ranging from 0.2 to 3%, depending on various factors such as species, origin, cultivation methods, and harvesting conditions [10,11,12]. For instance, while the average verbascoside content in Cistanche tubulosa Wight, also known as “desert ginseng”, can reach 4.75%, it is only 0.23% in Cistanche deserticola and Cistanche sinensis G. Beck [13]. Consequently, research efforts have focused on the synthesis and extraction of verbascoside. Although chemical synthesis techniques have been achieved, the complex steps, low extraction yields, and high costs currently hinder large-scale production [14]. Therefore, verbascoside derived from plant sources remains the dominant market option.

Extraction is an important step in isolating bioactive compounds from plants. To effectively obtain natural verbascoside, heated reflux extraction was applied [15], but it was often time-consuming, and continuous high-temperature heating can lead to the degradation of verbascoside. In pursuit of verbascoside extraction efficiency, methods like microwave-assisted extraction [12], ultrasound-assisted extraction (UAE) [10], fermentation treatment [15], and centrifugal extraction [16], have gradually replaced traditional techniques. Among these methods, UAE stands out as one of the most widely adopted technology and has demonstrated superior efficiency for polyphenol-rich extracts [17]. Its advantage lies in the potent cavitation effect induced by ultrasonic radiation pressure, which reduces the force between the target extract and the sample, facilitating solid–liquid separation [3]. Although UAE has proven its effectiveness, advancements in extraction technology have given rise to wall-breaking extraction (WBE), a method promising even greater efficiency. Recently, driven by the significant demand for verbascoside-rich extract (VRE) from plants, WBE technology has gained favor and is widely used in the food and pharmaceutical industries. This technique maximizes the retention of active ingredients from the natural plant without being heat-damaged, utilizing a minimal amount of solvent, resulting in a shortened extraction time, and enhanced efficiency [18]. As of now, there are limited studies exploring the impact of the widely-used UAE and innovative WBE on phenolic compounds.

Conventional extraction processes for extracting active substances from natural sources utilized traditional solvents such as acetone, styrene, chloroform, and cyclohexane. However, most of these solvents are toxic and volatile, and cause environmental pollution. In green extraction methods, the use of environmentally friendly and biodegradable green solvents has been extensively researched in order to avoid the use of toxic solvents and reduce extraction times. To this end, deep eutectic solvents (DESs) could potentially replace traditional solvents [19]. DESs are a new type of solvent that is both green and sustainable. Therefore, DESs have attracted increasing attention due to their low toxicity, enhanced environmental compatibility, and reduced cost [20].

The development of the olive oil industry has produced many byproducts, including pomace, wastewater, olive branches, and olive leaves. Among these byproducts, the Chinese olive industry produces about 600,000 tons of *Olea europaea* leaves every year, but these leaves are discarded or burned because of the lack of utilization channels [21,22]. Previous reports on olive have indicated that olive leaves are rich in oleuropein, verbascoside, hydroxytyrosol, flavonoids, and other phenolic compounds, which have attracted widespread attention due to their unique biological activities [22,23]. Among these various active ingredients, verbascoside is one of the most common, and the sustainable recovery of such valuable compounds from *O. europaea* leaves (presently underexploited) may find high-value applications in food and pharmaceutical products. Thus, VREs have great potential for integration into the food industry [23,24]. However, research in China and internationally has focused chiefly on active substances found in *O. europaea* leaves—the total phenols, flavonoids, and oleuropein—while verbascosides have not been sufficiently studied, especially in Chinese *O. europaea* cultivars.

The present study extracted polyphenolic compound verbascoside from olive leaves grown in China, using a UAE and WBE techniques with a DES consisting of choline chloride and ethylene glycol in a 1:2 molar ratio. This approach aimed to enhance the extraction efficiency of the phenolic compound verbascoside while minimize environmental contamination. The DES’s moisture content, liquid–solid ratio, and extraction time were optimized by employing the response surface methodology (RSM) with a Box–Behnken design (BBD). Subsequently, the total phenolic content (TPC) and phenolic compounds were determined by the Folin–Ciocalteu method and high-performance liquid chromatography (HPLC), respectively. This study specifically analyzed the effects of olive variety, leaf age, and extraction method on the main phenolic components. Additionally, the antioxidant activity of the VRE was investigated using two common radical scavenging assays: ferric 2,2′-azino-bis-3-ethylbenzthiazoline-6-sulphonic acid assay (ABTS^•+^ assay) and 1,1-diphenyl-2-picrylhydrazyl assay (DPPH^•^ assay). Furthermore, to evaluate the anticancer potential of extracts obtained through various extraction techniques and from different olive varieties, the cell viability of esophageal cancer Eca-109 cells (Eca-109) and a normal esophageal cell line (Het-1A) was assessed using the Cell Counting Kit-8 (CCK-8) assay.

## 2. Results and Discussion

### 2.1. Single-Factor Experiment Analysis

#### 2.1.1. Effects of Extraction Time on the TPC and VAY

**Figure 1:**
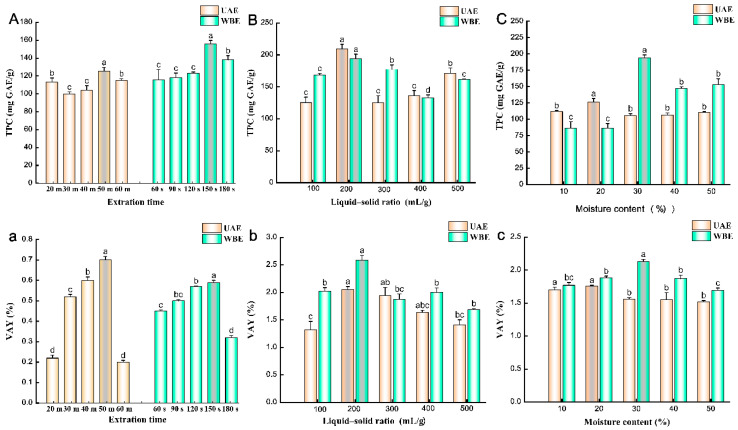
Effects of different (**A**,**a**) extraction times, (**B**,**b**) liquid–solid ratios, and (**C**,**c**) moisture content on TPC and VAY of UAE and WBE. UAE, ultrasound-assisted extraction; WBE, wall-breaking extraction. Different lowercase letters indicate significant differences at *p* < 0.05.

The extraction time can affect the TPC. When maintaining a liquid–solid ratio of 100 mL/g and a DES moisture content of 30%, Figure 1A illustrates that, in ultrasonic-assisted extraction (UAE), as the extraction time increased from 20 to 60 min, the TPC gradually rose from 99.67 to 125.48 mg GAE/g, peaking at 50 min. In the case of WBE, as the extraction time varied from 60 to 180 s, the TPC also increased from 115.49 to 155.87 mg GAE/g, with a maximum at 150 s. Similarly, as the extraction time was adjusted to ranges of 20–50 min for UAE and 60–180 s for WBE, the verbascoside extraction yield (VAY) of both methods exhibited an upward trend with increasing extraction time (Figure 1a). Specifically, the maximum yields of UAE and WBE were 0.70 and 0.59%, respectively. However, further extending the extraction time to 60 min for UAE and 180 s for WBE resulted in significant decreases in VAYs. This suggests that excessive extraction time can have a detrimental effect on VAY and may lead to the degradation of verbascoside, echoing findings from previous studies on extracting verbascoside from *Rehmannia* root [10]. Therefore, based on our optimization experiments, the recommended times for UAE and WBE are 50 min and 150 s, respectively.

#### 2.1.2. Effects of Liquid–Solid Ratios on the TPC and VAY

The liquid–solid ratio is one of the key factors influencing the TPC. With extraction times stabilized at 50 min for UAE and 150 s for WBE, and a DES moisture content of 20%, Figure 1B shows that, as the liquid–solid ratio increased from 100 to 200 mL/g, the TPC fluctuated between 125.26 and 209.63 mg GAE/g for UAE and 133.14 to 193.80 mg GAE/g for WBE, respectively, both reaching a peak at 200 mL/g. This is possibly due to the increased contact area between the matrix and the solvent, allowing for the greater diffusion of polyphenols from the intracellular matrix into the solvent [25]. However, further increasing the liquid–solid ratio from 200 to 500 mL/g led to a decrease in TPC to 125.27 mg GAE/g, suggesting that excessive extraction solvent may reduce the dissolution rate of phenolic compounds. The VAY exhibited a similar trend, with the liquid–solid ratios investigated ranging from 100 to 500 mL/g (Figure 1b). Specifically, VAYs increased significantly from 100 to 200 mL/g for both the UAE and WBE processes, also reaching a maximum at 200 mL/g, but then sharply declined in the range of 300–500 mL/g, dropping to 1.32% for UAE and 1.69% for WBE. This explanation is supported by the liquid–solid ratios used in the preparation of traditional Chinese medicine decoctions [4]. Thus, based on the optimization experiment, a liquid–solid ratio of 200 mL/g was selected.

#### 2.1.3. Effects of Moisture Contents on the TPC and VAY

The moisture content is one of the most significant factors affecting DES properties, such as viscosity and polarity [26]. As depicted in Figure 1C, during the UAE and WBE processes, with the liquid–solid ratio fixed at 100 mL/g and extraction times of 50 min and 150 s, respectively, varying moisture contents significantly impact TPC. In UAE, a DES moisture content of 20% resulted in a maximum TPC of 126.41 mg GAE/g, while other moisture contents had little effect on TPC. Conversely, in WBE, TPC was more responsive to changes in DES moisture content. As the moisture content increased from 10 to 30%, TPC gradually increased and peaked at 194.39 mg GAE/g. This suggests that an appropriate moisture content in DES can diminish solvent viscosity and enhance mass transfer efficiency, thereby accelerating the polyphenol extraction rate [27]. Studies have revealed that the density of DES is generally higher than water [28], and, as the moisture content of choline chloride–ethylene glycol (1:2 molar ratio) DES increases, its viscosity decreases [29]. Within the 20 to 30% moisture range, the highest VAYs of 1.76 and 2.13% were achieved for UAE and WBE, respectively (Figure 1c). Nevertheless, as the moisture content surpassed 30% and approached 50%, the extract yield progressively declined to 1.52 and 1.69% for UAE and WBE, respectively. Given that verbascoside is a water-soluble secondary metabolite, excess extraction solution may disrupt hydrogen-bonding interactions, thereby constraining the interplay between DES and target components [2,26]. Consequently, based on the optimization experiment, DES moisture contents of 20 and 30% were selected for UAE and WBE, respectively.

### 2.2. Optimization Experiment of RSM Analysis

#### 2.2.1. Extraction Model Analysis

After obtaining the single-factor results, a Box–Behnken Design (BBD) implemented with RSM software (Design-Expert 10 software (State-Ease Inc., Minneapolis, MN, USA)) was utilized to optimize three main factors: the extraction time, liquid–solid ratio, and moisture content in DES. The purpose was to determine the optimal conditions for TPC and VAY as response values in both UAE and WBE. According to the BBD model, three factors and three levels (−1, 0, and 1) were established (Table 6). The interactive effects of these factors on TPC and VAY were evaluated through 17 experimental runs (Table 1). In UAE, TPC ranged from 132.12 to 202.43 mg GAE/g, while, in WBE, it varied between 91.97 and 212.98 mg GAE/g. Similarly, VAY fluctuated between 1.21–1.49% in UAE and 0.94–1.29% in WBE. The quadratic model for TPC and VAY revealed significant linear and quadratic relationships, which were captured in Equations (1)–(4), used to predict TPC and VAY (Y) for both UAE and WBE:

Y_UAE-TPC_ = 199.68 + 7.24X_1_ + 4.58X_2_ − 4.13X_3_ + 11.97X_1_X_2_ + 3.48X_1_X_3_ − 6.26X_2_X_3_ − 25.67X_1_^2^ − 15.35X_2_^2^ − 13.06X_3_^2^ (1)

Y_UAE-VAY_ = 1.45 + 0.0238X_1_ + 0.0092X_2_ + 0.0056X_3_ − 0.0158X_1_X_2_ − 0.0405X_1_X_3_ − 0.0413X_2_X_3_ − 0.0726X_1_^2^ − 0.069X_2_^2^ − 0.1336X_3_^2^ (2)

Y_WBE-TPC_ = 205.46 + 1.06X_1_ − 0.34X_2_ + 3.94X_3_ + 9.93X_1_X_2_ + 4.56X_1_X_3_ + 12.64X_2_X_3_ − 35.77X_1_^2^ − 50.35X_2_^2^ − 44.17X_3_^2^ (3)

Y_WBE-VAY_ = 1.29 − 0.0287X_1_ + 0.0515X_2_ + 0.0115X_3_ + 0.0155X_1_X_2_ − 0.0243X_1_X_3_ − 0.0436X_2_X_3_ − 0.0696X_1_^2^ − 0.0904X_2_^2^ − 0.1504X_3_^2^ (4)

Based on ANOVA, the adequacy and goodness-of-fit of the quadratic regression model are listed in Table 2 A high F-value and a low *p* value (*p* < 0.05) in the models showed that the response variable was more significant [30]. As can be seen from Table 2, the models for TPC and VAY are significant in both UAE and WBE, with *p* < 0.0001; the F-values for TPC were 26.08 and 101.64, respectively, and the F-values for VAY were as high as 357.36 and 154.48, respectively, illustrating the high significance of both models. For UAE, the coefficients of determination (R^2^) for TPC and VAY were 0.971 and 0.9978, respectively, with adjusted coefficients of determination (Adj.R^2^) of 0.9338 and 0.995, respectively. Similarly, for WBE, the R^2^ for TPC and VAY were 0.9924 and 0.995, respectively, with Adj.R^2^ values of 0.9826 and 0.9885, respectively, demonstrating the high reliability of the model. Furthermore, the lack of fit test was not significant (*p* > 0.05), indicating that the regression equation accurately captures the impact of the three factors on TPC and VAY, effectively optimizing the extraction process for both UAE and WBE. The coefficients of variation (C.V.%) for TPC were 3.01 and 3.86% for UAE and WBE, respectively, while those for VAY were 0.4937 and 1.06%, respectively, confirming the good reproducibility and reliability of the experimental results.

Finally, in terms of factor effects, the linear effects of the extraction time (X_1_), liquid–solid ratio (X_2_), and moisture content of DES (X_3_) on TPC were more significant in WBE than in UAE (Table 2). While the interaction terms of X_1_X_2_ and X_2_X_3_ impacted TPC in both methods, only X_1_ and X_2_ had significant linear effects in UAE, with no effect in WBE. For VAY, all linear effects (X_1_^2^, X_2_^2^, X_3_^2^) and interaction terms were highly significant (*p* < 0.0001), with the linear term of extraction time (X_1_) in UAE and the liquid–solid ratio (X_2_) in WBE displaying particularly significant.

#### 2.2.2. Response Surface Analysis

**Figure 2:**
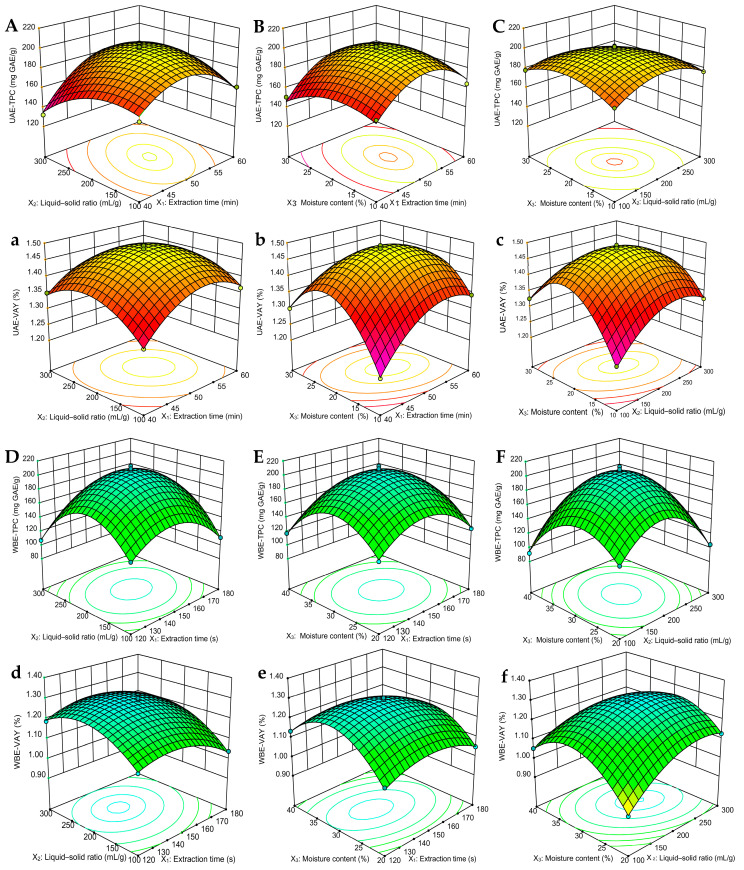
Three-dimensional response surface figures for UAE and WBE: (**A**,**a**,**D**,**d**) extraction time and liquid–solid ratio; (**B**,**b**,**E**,**e**) extraction time and moisture content; and (**C**,**c**,**F**,**f**) liquid–solid ratio and moisture content on the TPC and VAY of olive leaves. TPC, total phenolic content; VAY, verbascoside yield.

Response surface plots directly show the interactions between factors, and, during the change process, the steeper the response surface, the more obvious the impact [31]. During the UAE and WBE process, a three-dimensional (3D) response surface can more intuitively display any two-factor interaction of extraction parameters on the TPC and VAY. When other factors are fixed, the greater the interaction of extracted factors is, the steeper the figure will be [25]. In UAE, Figure 2A–C demonstrate that, under the interaction conditions of X_1_X_2_ and X_2_X_3_, the 3D surface is steeply inclined, indicating a significant impact of the UAE method on TPC. Specifically, when the time is set at 50 min, the liquid–solid ratio is 200 mL/g, and the moisture content is 20%, the UAE-TPC achieves a peak value of approximately 200 mg GAE/g (Figure 2A,C). However, the interaction effect of X_1_X_3_ on UAE-TPC is minimal and does not reach a significant level (Figure 2B). Concurrently, the interactions among X_1_X_2_, X_1_X_3_, and X_2_X_3_ exhibit an observable influence on the VAY (Figure 2a–c). Notably, the interplay between the moisture content in DES, the extraction time, and the liquid–solid ratio attains a highly significant level, yielding a pronounced curve (Figure 2b,c), within which the UAE-VAY varies between 1.21 and 1.49%.

Similarly, in WBE, Figure 2D–F reveal that, under stable conditions of other factors, the interactions X_1_X_2_ and X_2_X_3_ continue to exert a substantial influence on TPC. Specifically, when the moisture content in DES was set at 30%, the extraction time was 150 s, and the liquid–solid ratio was 200 mL/g, WBE-TPC attained its peak, presenting an extremely steep surface (Figure 2D,F). Here, WBE-TPC varied within the range of 140–200 mg GAE/g, while X_1_X_3_ exhibits a minor interaction effect on WBE-TPC. Concurrently, the WBE-VAY displayed greater volatility, ranging from 0.94 to 1.30%. As depicted in Figure 2d–f), the interactions among X_1_X_2_, X_1_X_3_, and X_2_X_3_ are noteworthy. Although the moisture content (X_1_) in DES, as a linear term, has a lower impact on the yield compared to the extraction time (X_2_) and liquid–solid ratio (X_3_), when combined, their interactions become more significant. Notably, at 150 s, the combination of moisture content with both the liquid–solid ratio (X_1_X_3_) and extraction time (X_2_X_3_) results in a highly significant impact on the extraction yield (Figure 2e,f). The findings presented in the 3D figures were also in harmony with the data summarized in Table 3. Therefore, the 3D plot and ANOVA analysis (Table 2) indicated that the most important factor influencing the TPC and VAY in UAE and WBE was extraction time, followed by the liquid-to-solid ratio, and, finally, the moisture content in DES.

#### 2.2.3. Optimization of Extraction Conditions

To verify whether there was any deviation in the experimental results regarding TPC and VAY, further verification experiments were performed to obtain the optimal conditions (Table 3). According to the optimization experimental results, the optimized conditions for TPC and VAY in UAE are as follows: an extraction time of 51.14 and 55 min, a liquid–solid ratio of 190.20 and 200 mL/g, and a moisture content of 18.76 and 19%. For simplicity of operation, high efficiency, and energy saving, the actual operating conditions are set as follows: an extraction time of 55 min, a liquid–solid ratio of 200 mL/g, and a moisture content of 20%. Under these conditions, the highest yields are 206.23 ± 0.58 mg GAE/g and 1.59 ± 0.04%, respectively. In WBE, the optimized conditions for TPC and VAY, based on the experimental results, are as follows: an extraction time of 150.31 and 140 s, a liquid–solid ratio of 190.92 and 230 mL/g, and a moisture content of 30%. For practical application, the actual extraction time was set to 140 s, the moisture content was set to 30%, and the liquid–solid ratio was maintained at 210 mL/g. Under these conditions, the optimal values of TPC and VAY were 210.69 ± 0.97 mg GAE/g and 1.33 ± 0.2%, respectively.

### 2.3. Quantitative Analysis of Phenolic Compounds

**Figure 3:**
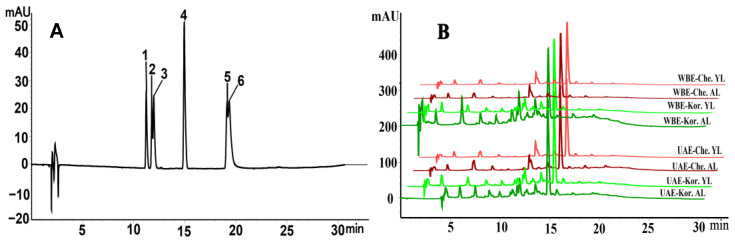
High-performance liquid chromatography (HPLC) chromatogram of the standard (**A**) and the representative sample (**B**) from olive leaves for phenolic compounds; Che, Chemlal variety; Kor, Koroneiki variety; AL, aged leaves; YL, young leaves.

Six major phenolic compounds were identified and quantified in the olive leaves, and the HPLC technique reported from leaves of Liangshan *Olea europaea* in our previous work was used [21]. As shown in Table 4, the HPLC chromatogram of the representative sample (Figure 3B) was obtained under optimized conditions by matching the peak retention time of the sample to the phenolic compound standards (Figure 3A). The quantification of six phenolic compounds in old and young leaves of five major olive cultivars, extracted using both the UAE and WBE methods, is shown in Table 5.

Table 5 shows that, in UAE and WBE, TPC fluctuations among various olive leaf types were significant, ranging from 108.33 to 180.72 mg GAE/g in UAE, and from 59.01 to 217.40 mg GAE/g in WBE. Notably, in both methods, young leaves accumulated significantly higher TPC levels compared to old leaves. Among the five cultivated varieties, during the UAE process, the young leaves of the Koroneiki and Chemlal varieties exhibited the highest TPC levels, at 180.72 and 171.09 mg GAE/g, respectively. In the WBE process, the young leaves of the Arbosana and Chemlal varieties had the highest TPC content, reaching 217.40 and 173.29 mg GAE/g, respectively. Conversely, during UAE, the Arbequina and Picual varieties demonstrated the lowest TPC in old leaves, with levels as low as 128.39 and 108.33 mg GAE/g, respectively. In the WBE process, the old leaves of the Arbequina and Koroneiki varieties showed the lowest TPC levels, at 72.56 and 59.01 mg GAE/g, respectively. This variation in variety and leaf age aligns with our previous findings on TPC in Liangshan olive leaves [26].

HPLC analysis further revealed significant differences in the changes of the four main phenolic components among varieties and leaf ages (Table 5). For rutin, concentrations ranged from 0.05 to 0.8% in UAE and from 0.01 to 0.54% in WBE. For verbascoside, the range was from 0.30 to 3.51% in UAE and from 0.43 to 2.89% in WBE. Luteolin-4′-*O*-glucoside concentrations varied from 0.19 to 0.66% in UAE and from 0.16 to 0.46% in WBE. Oleuropein levels ranged from 0.97 to 7.46% in UAE and from 1.32 to 6.09% in WBE. However, the contents of luteolin and quercetin in UAE were relatively low, both below 0.07%. Unlike in UAE, the luteolin content was higher in WBE, reaching a maximum value close to 0.020%. Quercetin, on the other hand, exhibited extreme instability and was only detected in extremely low and unstable amounts in the Chemlal and Picholine varieties. Regarding variety changes, the Chemlal and Koroneiki varieties particularly stood out in UAE, while the Chemlal and Arbosana varieties excelled in WBE, accumulating notably higher levels of verbascoside, oleuropein, luteolin, and quercetin. In comparison, the Koroneiki variety had relatively high levels of rutin and luteolin-4′-*O*-glucoside in UAE, whereas, in WBE, the Arbosana variety showed higher contents of rutin, luteolin-4′-*O*-glucoside, and luteolin compared to other varieties. The observed significant variations in oleuropein content among different leaf ages of seven Italian olive varieties in this study were consistent with previous findings [32]. In summary, both UAE and WBE significantly alter the phenolic profile of olive leaves. Generally, WBE resulted in higher TPC and rutin levels, while UAE tended to produce marginally elevated concentrations of other phenolic compounds. This may be because, during the WBE process, the internal and external pressure difference facilitated the expansion and rupture of the plant cell-wall [33], making rutin easier to release into the solvent. However, UAE destroyed the plant cell structure and prolonged the extraction time, which enhanced solvent permeability, making phenolic substances easier to dissolve [34]. However, these hypotheses require further experimental verification.

### 2.4. Antioxidant Activity Assay

Based on a quantitative analysis of phenolic compounds in the leaves of five primary cultivated varieties at various stages, an evaluation of the antioxidant activity of VRE was conducted. This assessment focused on the young leaves of the representative varieties Chemlal and Koroneiki, extracted using UAE and WBE methods. For benchmarking purposes, verbascoside purified to 50% was also tested.

**Figure 4:**
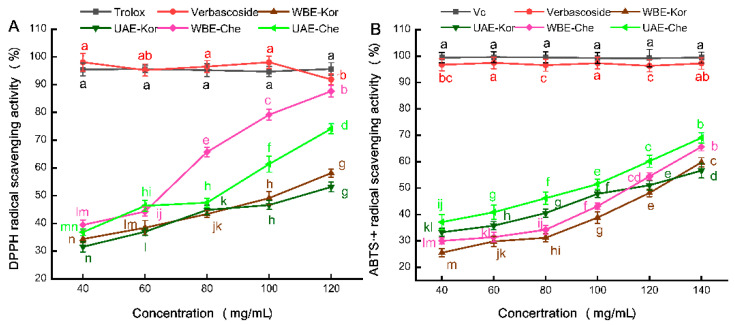
Antioxidant activities of VREs from the Chemlal (Che) and Koroneiki (Kor) varieties extracted by UAE and WBE. (**A**) DPPH• radical scavenging activity; (**B**) ABTS•+ radical scavenging activity. VRE, verbascoside-rich extract; Vc, vitamin C. Results are expressed as the percentage of control group growth for each data point and represent the mean (±SE) of three independent experiments; Diverse letters correspond to disparate samples or varying concentrations, which exhibit significant differences.

To assess the antioxidant potency of these VREs, we employed two widely used in vitro methods: DPPH^•^ and ABTS^•+^ assays. The DPPH^•^ assay measures the reduction in purple DPPH^•^ to 1,1-diphenyl-2-picrylhydrazyl, while the ABTS^•+^ assay determines the capacity of antioxidants to reduce blue/green ABTS^•+^ radicals [35]. Both methods are preferred for their simplicity and have been widely used in evaluating the antioxidant potential of plant extracts [36,37]. A comparative analysis, using both assays, is presented in Figure 4A,B.

#### 2.4.1. DPPH^•^ Assay

Figure 4A illustrates that the control Trolox exhibited a similar DPPH^•^ scavenging capacity to verbascoside, reaching approximately 95%. However, a notable difference was observed in the DPPH^•^ scavenging abilities of the UAE- and WBE-extracted VREs. Specifically, WBE-Che demonstrated superior scavenging activity compared to UAE-Che at concentrations ranging from 80 to 120 mg/mL. Higher EC_50_ values indicated lower antioxidant activity, and the EC_50_ value (57 mg/mL) of WBE-Che was lower than that of UAE-Che (69 mg/mL). In a comparable study utilizing maceration or methanolic extraction, DPPH^•^ assays revealed variations in the antioxidant potency of verbascoside [38]. In the current study, the Koroneiki extracts displayed the lowest antioxidant activity, with similar EC_50_ values of UAE-Kor (114 mg/mL) and WBE-Kor (112 mg/mL) were close. This could be attributed to the higher TPC content in WBE, as a significant correlation between antioxidant capacity and TPC has been documented [39], implying that phenolic compounds were the primary contributors to WBE-Che’s antioxidant attributes.

#### 2.4.2. ABTS^•+^ Assay

As shown in Figure 4B, verbascoside demonstrates an exceptionally strong ABTS^•+^ free radical scavenging capability, reaching approximately 97% compared to the control Vc. However, the ABTS^•+^ free radical scavenging efficacy of VREs was significantly influenced by both the extraction method and the olive variety. For the Chemlal variety, UAE-Che appeared to exhibit superior antioxidant potential against the ABTS^•+^ radical compared to WBE-Che, with the EC_50_ value of UAE-Che (99 mg/mL) being much lower than that of WBE-Che (113 mg/mL). This suggests a correlation between the verbascoside content, determined by the extraction technique, and its antioxidant activity [30]. For the Koroneiki variety, the UAE- and WBE-Kor extracts demonstrated lower activity, showing a good linear dose–effect relationship within the 40–140 mg/mL concentration range. At the same time, close EC_50_ values were observed for both UAE-Kor (134 mg/mL) and WBE-Kor (132 mg/mL). These results highlight the strong ABTS^•+^ free radical scavenging capability of UAE-Che, indicating that the scavenging efficacy of VREs is not only variety-dependent but also significantly influenced by the extraction method [39,40]. This could be due to the rich verbascoside, oleuropein, luteolin, and quercetin, presented in the leaves of the Chemlal variety, which played a crucial role in ABTS^•+^ scavenging free radicals during the UAE process [41].

### 2.5. Anticancer Activity Assay

The anticancer effects of VREs from the Chemlal and Koroneiki varieties extracted using UAE and WBE were investigated in human esophageal cell lines. Het-1A and Eca-109 were used to determine the cell viability at 25 and 50 µg/mL for 24 h using the CCK-8 assay, and the results were compared with the control (verbascoside standard drug).

**Figure 5:**
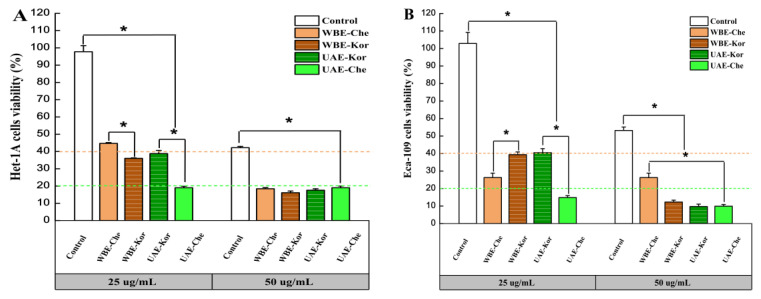
Anticancer activities of VREs from the Chemlal (Che) and Koroneiki (Kor) olive varieties extracted using UAE and WBE at 25 and 50 µg/mL for 24 h. (**A**) Het-1A cell viability; (**B**) Eca-109 cell viability. Results are expressed as the percentage of control group growth for each data point and represent the mean (±SE) of three independent experiments; * *p* ≤ 0.001 (ANOVA Duncan’s test). VRE, verbascoside-rich extract; UAE, ultrasound-assisted extraction; WBE, wall-breaking extraction.

As shown in Figure 5A,B, at 25 µg/mL, the cell viability of all VRE-treated cells decreased significantly compared to the control, indicating that verbascoside had no direct inhibitory effect on the grow of Het-1A and Eca-109 cells. Previous studies demonstrated that verbascoside did not exhibit any cytotoxic effects on human dermal papilla cells at a concentrations of 0.98 to 500 µg/mL [42]. Under the same concentration, the Chemlal extracts promoted the cell viability of Het-1A, which the WBE-Che extracts (44.69%) caused to proliferate more markedly than UAE-Che (18.99%). However, inhibition activities appeared in Eca-109, against which UAE-Che (14.83%) had a stronger inhibitory effect than WBE-Che (26.27%). Interestingly, Koroneiki extracts at 25 µg/mL showed a similar cell viability of about 40% in Het-1A and Eca-109 cells. In a similar study, isolebascoside and verbascoside extracts showed rather weak inhibitory activity against AChE, yet strong cytotoxic activity against MCF-7 cells [43]. This result demonstrates that the same extract has different inhibitory effects on different cell lines.

At 50 µg/mL, the VRE strongly inhibited the cell growth of Het-1A and Eca-109, and the cell growth in the control also dropped to 42.21 and 53.17%, respectively (Figure 5A,B), suggesting that the antiproliferative effect strengthened with an increase in the concentration of the extract [44]. Another study found that treating Chinese hamster lung fibroblast V79 cells with verbascoside resulted in a cytotoxic effect at concentrations of 50 µg/mL and higher as measured by neutral red uptake [12]. In the present study, both cell lines suffered varying degrees of growth inhibition in all samples, with Eca-109 being more sensitive to high concentrations than Het-1A. Remarkably, WBE-Che extracts had a stronger inhibitory effect on Het-1A (18.44%) than Eca-109 (26.27%), indicating that a high concentration of WBE-Che VRE was extremely detrimental to the growth of Het-1A. Similar effects were previously demonstrated in a study that found that verbascoside in *Plantago lagopus* L. had strong cytotoxic activity against the MCF-7 cell line, and cell apoptosis occurred when treated with 50–100 µg/mL verbascoside [45]. Collectively, the results indicated that 25 µg/mL was a suitable concentration to promote Het-1A activity and inhibit Eca-109 growth, and, at this concentration, WBE-Che VRE was more efficient than that obtained from the Koroneiki cultivar. However, further experiments are necessary to explore and verify these findings.

## 3. Materials and Methods

### 3.1. Materials and Chemicals

The five olive varieties were grown at the Hongfu Olive Base in Nanchong City, Sichuan Province, China, and were collected in December, 2022. Among them, the young leaves of the Arbeqoina variety were used for optimization analysis. The leaves were washed and dried at 50 °C, and then ground into powder using a high-speed grinder (FD-15-T-350A, Yushuo Chinese herbal medicine high-speed crusher, Shanghai, China). The powder was sieved to obtain particle sizes < 0.3 mm, and stored at 4 °C for further analysis by UAE and WBE process.

Methanol and acetonitrile were of chromatography grade (Thermo Fisher Scientific Co., Ltd., Shanghai, China); HPLC-grade (≥98%) rutin, oleuropein, verbascoside, luteolin, quercetin, and luteolin-4′-*O*-glucoside were purchased from Must Biotechnology (Chengdu, China); Folin–Ciocâlteu’s reagent, choline chloride, and ethylene glycol were of analytical grade (Hengxing Chemical Reagent Co., Ltd., Tianjin, China). In addition, ultrapure water was used for the extraction and analysis of verbascoside using the UPT-T-101 UPT ultrapure water machine (0.22 mm) (Chengdu Ultrapure Water Technology Co., Ltd., Chengdu, China).

### 3.2. Preparation of DESs

DESs were prepared according to the method of Dardavila et al. [46] with minor modifications. In this experiment, DES was prepared from choline chloride and ethylene glycol by mixing the reagents in a 1:2 molar ratio, and then stirring the mixture under ultrasonic vibration at 210 W power and 40 °C temperature until a uniform transparent liquid formed, which the DES required. Then, the DES was sealed in glass flasks at room temperature (25 °C) for further trials.

### 3.3. Experimental Design Using UAE and WBE

#### 3.3.1. Single-Factor Experiment

Firstly, dried powder (1 g) of young leaves was mixed with different moisture contents of DES in a 1000 mL tube and extracted for various durations using different liquid–solid ratios. Two extraction methods, UAE and WBE, were then employed. The extraction procedures, with consistent influencing factors across methods, were as follows:

(1) UAE methods: UAE was performed using an ultrasonic bath (KH7200DE, 40 kHz, Kunshan Hechuang Co., Ltd., Kunshan, China) operating at a constant ultrasonic frequency of 40 kHz and power of 320 W. Maintaining a temperature of 25 ± 2 °C, we studied three key factors separately: ultrasonic extraction time (20, 30, 40, 50, and 60 min), moisture content in DES (10, 20, 30, 40, and 50%), and liquid–solid ratio (100, 200, 300, 400, and 500 mg/mL) on TPC and VAY.

(2) WBE methods: An RBM-769S food multifunctional wall-breaking machine (HATTIECS, Zhongshan Huiren Electric Co., Ltd., Zhongshan, China) was utilized for extraction. With an operating temperature of 25 °C and power fixed at 800 W, we evaluated the effects of wall-breaking time (60, 90, 120, 150, and 180 s), moisture content in DES (10, 20, 30, 40, and 50%), and liquid–solid ratio (100, 200, 300, 400, and 500 mg/mL) on TPC and VAY.

Finally, the extraction solution underwent centrifugation at 2795× *g* for 10 min (TGL-16.5M, Shanghai Lu Xiangyi Centrifuge Instrument Co., Ltd., Shanghai, China). The supernatant was collected for optimization analysis and also freeze-dried to obtain VRE. The resulting VRE was sealed and stored at −18 °C for activity analysis.

#### 3.3.2. RSM Optimization Experiment

According to single-factor experiment result, the BBD model with RSM was used in Design-Expert 20 software to optimize the UAE and WBE extraction conditions for TPC and VAY. The BBD was conducted with three independent variables (X_1_, extraction time; X_2_, liquid–solid ratio; and X_3_, moisture content in DES) and three levels (−1, 0, and 1), and the TPC and VAY was taken as the response value. The coding levels and actual levels of the three factors are shown in Table 6.

### 3.4. HPLC Analysis of Phenolic Compounds

Qualitative and quantitative analyses of phenolic compounds were conducted using an HPLC instrument (Agilent (Santa Clara, CA, USA) 1260 HPLC) connected to a C18 reversed-phase column (Agilent (Santa Clara, CA, USA), ZORBAX Eclipse XDB-C18, 5.0 µm, 150 × 4.6 mm). The column temperature remained constant at 30 °C, and the injection volume was 10 µL. Eluent A (acetonitrile) and eluent B (water/acetic acid, 99.8/0.2 *v*/*v*) were used as mobile phases at a flow rate of 0.8 mL/min. The detection wavelength was 240 nm. The mobile phase was: 10–16% A from 0–5 min, 16–25% A from 5–10 min, 25–38% A from 10–25 min, and 38–10% A from 25–30 min. The extraction yields of phenolic compound were determined using the standard calibration curve, and the results were calculated according to the following equation:

$$\mathrm{Yield}\;(\%)=\frac{\mathrm{mass}\;\mathrm{dried}\;\mathrm{crude}\;\left(g\right)}{\mathrm{mass}\;\mathrm{of}\;\mathrm{olive}\;\mathrm{leaf}\;\mathrm{powder}\;\left(g\right)}\times 100$$

### 3.5. Determination of Total Phenolic Content (TPC)

The TPC of extracts from Chinese olive leaves was determined using the Folin–Ciocalteu colorimetric method with slight modifications. Gallic acid (0.005–0.70 mg/mL) was used for the calibration of a standard curve. The absorbance was measured at 760 nm with a microplate reader (Thermo Multiskan GO, East Lyme, CT, USA). The results are expressed as milligrams of gallic acid equivalents per gram of extract (mg GAE/g).

### 3.6. Determination of Antioxidant Activity

The antioxidant activity was analyzed using DPPH^•^ and ABTS^•+^ kits (Grace Bioengineering Institute, Suzhou, China), which mainly analyzed the free radical scavenging activity of VRE. The half-effective concentration (EC_50_) value was the sample concentration required for the 50% scavenging of free radicals and was determined based on a plot between the inhibition rate and the concentration.

#### 3.6.1. DPPH^•^ Radical Scavenging Activity

The DPPH^•^ scavenging activity was determined using a DPPH^•^ assay. Briefly, 150 μL of different concentrations of VRE (40, 60, 80, 100, and 120 mg/mL) and 150 μL DPPH^•^ reagent (0.1 mM) was mixed in a 1.5 mL centrifuge tube. Subsequently, the reaction was measured after 30 min in the dark, and the absorption maximum was determined at 517 nm using a microplate reader (Thermo Multiskan GO, USA). The blank and the positive control consisted of 80% methanol and Trolox, respectively. The scavenging activity was calculated using the following equation:

$${\mathrm{DPPH}}^{•}\;\mathrm{radical}\;\mathrm{scavenging}\;\mathrm{activity}\;(\%)=1-(\frac{A_{\mathrm{test}}\;-\;A_{\mathrm{control}}}{A_{\mathrm{blank}}})\times 100$$

#### 3.6.2. ABTS^•+^ Radical Scavenging Activity

The ABTS^•+^ radical scavenging activity was evaluated using an ABTS^•+^ assay. First, 10 μL of different concentrations of the VRE (40, 60, 80, 100, 120, and 140 mg/mL) was mixed with 190 µL ABTS^•+^ reagent (0.1 mM) in a 1.5 mL centrifuge tube. The solution was placed in a dark environment for 6 min at room temperature (25 °C). Then, a 200 μL reaction solution was used to determine the absorbance at 734 nm with a microplate reader (Thermo Multiskan GO, USA). Anhydrous ethanol and vitamin C (Vc) served as the blank and the positive control, respectively. The ABTS^•+^ radical scavenging activities of the extracts were expressed as follows:

$${\mathrm{ABTS}}^{•+}\;\mathrm{radical}\;\mathrm{scavenging}\;\mathrm{activity}\;(\%)=1-(\frac{A_{\mathrm{test}}\;-\;A_{\mathrm{control}}}{A_{\mathrm{blank}}})\times 100$$

### 3.7. Determination of Antitumor Activity

#### 3.7.1. Cell Culture

The esophageal squamous cell carcinoma cell line (Eca-109) and normal esophageal epithelial cell line (Het-1A) were obtained from the College of Life Science of China West Normal University (Sichuan, China). The cells were dissolved in RPMI-1640 medium (CGM112.05, Cellmax Technology Co., Ltd., Beijing, China), which consisted of 10% fetal calf serum and 1% penicillin-streptomycin (CGM112.05). Cells were cultured at 37 °C in a 5% carbon dioxide (CO_2_) environment. When the cell density reached 80–90%, cells were sub-cultured at a ratio of 1:2.

#### 3.7.2. Cell Viability Assay

Cell viability was determined using CCK-8 kits (GBBIO Technologies Inc., Guangzhou, China) according to the manufacturer’s instructions. First, cells were counted and seeded in a 96-well plate with a volume of 100 μL at a density of 1 × 10^4^ cells/well. Then, the VRE was diluted with RPMI-1640 culture medium (CellMax CGM112.05, Sai Aomei Cell Technology Co., Ltd., Beijing, China) and prepared into samples of different concentrations (25 and 50 μg/mL). After incubation for 24 h, the CCK-8 experiment was performed. CCK-8 solution (10 µL) was added to each well and incubated for 2 h. The absorbance value was detected at 450 nm with a microplate reader (Multiskan FC). Eca-109 and Het-1A cells without sample treatment functioned as control groups. Cell viability was determined according to the following equations:

$$\mathrm{Cell}\;\mathrm{viability}\;(\%)=\frac{A_{\mathrm{sample}}\;-\;A_{\mathrm{control}}}{A_{\mathrm{control}}\;-\;A_{\mathrm{blank}}}\;\times 100\%$$

where A_sample_ is the absorbance of a well with cells, CCK-8 solution, and sample solution; A_blank_ is the absorbance of a well with medium and CCK-8 solution, without cells; and A_control_ is the absorbance of a well with cells and CCK-8 solution, without a sample solution.

### 3.8. Statistical Analysis

Design-Expert 10 software (State-Ease Inc., Minneapolis, MN, USA) with RSM was used to optimize the UAE and WBE processes and to determine the effects of their interactive effects on the response variables. Subsequently, data processing was conducted and figure drawings were created using Excel 2010 and SPSS Statistics 20.0 (IBM Corp., Chicago, IL, USA), and data were assessed using one-way analysis of variance (ANOVA). Finally, all experiments were independently repeated at least three times, with triplicate samples for each treatment, and results were displayed as the mean ± standard deviation (SD). Different letters in figures and tables indicated significant differences (*p* < 0.05).

## 4. Conclusions

In this study, to achieve a high-efficiency and environmentally friendly process for extracting phenolic component verbascoside from Chinese olive leaves, DES was utilized as the extraction agent. Based on optimization experiments, the optimal conditions for extracting TPC and VAY using UAE were determined to be as follows: extraction time of 55 min, a liquid–solid ratio of 200 mL/g, and a moisture content of 20%. These conditions yield a TPC of 206.23 ± 0.58 mg GAE/g and a VAY of 1.59 ± 0.04%. For WBE, the optimal conditions were as follows: extraction time of 140 s, a liquid–solid ratio of 210 mL/g, and a moisture content of 30%, resulting in a TPC of 210.69 ± 0.97 mg GAE/g and a VAY of 1.33 ± 0.2%. Under these conditions, significant TPC variations were observed among different olive leaf types and ages in both UAE and WBE, with young leaves generally accumulating a higher TPC than old leaves. Among the five main varieties, the Koroneiki and Chemlal varieties exhibited the highest TPC using UAE, while Arbosana and Chemlal had the highest using WBE. HPLC analysis revealed that WBE resulted in higher TPC and rutin levels, whereas UAE marginally increased other phenolic compounds, such as verbascoside, luteolin-4′-O-glucoside, oleuropein, luteolin, and quercetin. The antioxidant potency of VREs is significantly affected by extraction methods (UAE and WBE) and olive varieties. The Chemlal variety extracted using WBE showed stronger activity in the DPPH^•^ assay, whereas UAE was more effective in the ABTS^•+^ test, and the Koroneiki variety demonstrated the lowest activity. Additionally, the anticancer activity of different extracts against esophageal cancer cells was assessed using CCK-8 assays, and it was found that VREs significantly promoted Het-1A cell growth at 25 μg/mL for 24 h, whereas Eca-109 cells had a higher sensitivity to treatment with the Chemlal VRE. Especially at 50 μg/mL, the cell viability of both kinds of cells decreased noticeably, suggesting that the WBE-Che VRE possessed a powerful antioxidant ability and anti-proliferative efficiencies at a concentration of 25 μg/mL. In terms of energy efficiency, WBE was a relatively efficient method, and the Chemlal variety can serve as a potential source of verbascoside.

## Figures and Tables

**Table 1:** Box–Behnken design for the optimization of extraction conditions using UAE and WBE methods on TPC and VAY *.

Run		UAE					WBE			
	X_1_ (min)	X_2_ (mL/g)	X_3_ (%)	TPC (mg GAE/g)	VAY (%)	X_1_ (s)	X_2_ (mL/g)	X_3_ (%)	TPC (mg GAE/g)	VAY (%)
1	50 (0)	200 (0)	20(0)	150.69	1.48	150 (0)	200 (0)	30 (0)	195.64	1.29
2	60 (+1)	100 (−1)	20	164.36	1.37	120 (−1)	100 (−1)	30	130.79	1.12
3	50	100	30 (+1)	163.13	1.33	150	200	30	104.64	1.29
4	40 (−1)	100	20	132.12	1.29	150	200	30	91.97	1.29
5	40	300 (+1)	20	192.70	1.35	120	300 (+1)	30	129.85	1.19
6	50	300	10 (−1)	178.25	1.33	150	100	40 (+1)	117.41	1.05
7	50	200	20	201.68	1.48	180 (+1)	100	30	209.28	1.04
8	60	300	20	176.83	1.36	180	200	40	124.51	1.02
9	50	200	20	154.50	1.49	150	300	20 (−1)	207.32	1.13
10	60	200	10	202.43	1.34	150	100	20	128.73	0.94
11	60	200	30	175.54	1.27	120	200	40	111.38	1.13
12	50	100	10	200.59	1.23	150	200	30	128.70	1.30
13	40	200	30	201.02	1.30	180	200	20	118.42	1.05
14	40	200	10	164.26	1.21	120	200	20	107.44	1.07
15	50	200	20	161.27	1.48	150	300	40	202.08	1.07
16	50	200	20	178.15	1.48	150	200	30	129.39	1.27
17	50	300	30	164.51	1.25	180	300	30	212.98	1.17

* UAE, ultrasound-assisted extraction; WBE, wall-breaking extraction; X_1_, extraction time; X_2_, liquid–solid ratio; X_3_, moisture content in DES; TPC, total phenolic compound; VAY, verbascoside yield.

**Table 2:** Analysis of variance (ANOVA) for the experimental results obtained *.

Source	UAE				WBE			
	TPC		VAY		TPC		VAY	
	F-Value	*p*-Value	F-Value	*p*-Value	F-Value	*p*-Value	F-Value	*p*-Value
Model	26.08	<0.0001 ***	357.36	<0.0001 ***	101.64	<0.0001 ***	154.48	<0.0001 ***
X_1_-time	15.25	0.0059 *	100.86	<0.0001 ***	0.29	0.6061	44.75	0.0003 **
X_2_-ratio	6.10	0.0429 *	15.16	0.0059 **	0.03	0.8682	144.06	<0.0001 ***
X_3_-moisture	4.97	0.061	5.62	0.0495 *	4.00	0.0855	7.21	0.0313 *
X_1_X_2_	20.88	0.0026 *	22.37	0.0021 **	12.75	0.0091 *	6.55	0.0376 *
X_1_X_3_	1.76	0.2259	146.43	<0.0001 ***	2.69	0.1448	16.05	0.0051 *
X_2_X_3_	5.71	0.0483 *	152.07	<0.0001 ***	20.64	0.0027 *	51.61	0.0002 **
X_1_^2^	100.99	<0.0001 ***	486.17	<0.0001 ***	174.06	<0.0001 ***	138.42	<0.0001 ***
X_2_^2^	36.09	0.0005	444.94	<0.0001 ***	344.97	<0.0001 ***	233.26	<0.0001 ***
X_3_^2^	26.14	0.0014	1583.95	<0.0001 ***	265.47	<0.0001 ***	645.58	<0.0001 ***
Lack of Fit	2.74	0.1775 ns	1.91	0.2696	0.25	0.8581 ns	0.6349	0.6306 ns
R^2^	0.971		0.9978		0.9924		0.995	
R^2^adj	0.9338		0.995		0.9826		0.9885	
C.V.%	3.01		0.4937		3.86		1.06	

* Significant at *p* < 0.05; ** Significant at *p* < 0.001; *** Significant at *p* < 0.0001; ns, not significant (*p* > 0.05).

**Table 3:** Optimization conditions obtained using BBD *.

Factors	UAE				WBE			
	OC-TPC	AOC-TPC	OC-VAY	AOC-VAY	OC-TPC	AOC-TPC	OC-VAY	AOC-TPC
Time	51.14 min	55 min	55 min	55 min	150.31 s	140 s	140 s	140 s
Ratio (mL/g)	190.20	200	200	200	190.92	230	210	210
Moisture (%)	18.76	20	20	20	29.55	30	30	30
Yield	206.23 ± 0.58 mg GAE/g		1.59 ± 0.04%		210.69 ± 0.97 mg GAE/g		1.33 ± 0.2%	

* OC, optimization conditions; AOC, actual operating conditions.

**Table 4:** Analysis of phenolic compounds derived from Chinese olive leaves *.

Compound	Linear Calibration Range (mg/mL)	Calibration Equation	Regression Coefficient (R^2^)	Retention Time (min)
TPC	0.005–0.70	y = 0.1698x + 0.1622	0.9994	—
1	0.001–0.0045	y = 13,217x + 17.106	0.9961	11.224
2	0.01–0.09	y = 5073.7x + 2.85	0.9976	11.772
3	0.005–0.035	y = 23,387x + 5.4543	0.9923	11.96
4	0.05–0.35	y = 16,378x − 77.557	0.9998	14.942
5	0.0015–0.005	y = 31,410x + 9.419	0.9962	19.128
6	0.0015–0.005	y = 26,441x + 8.913	0.9923	19.347

* x: concentration (mg/mL); y: peak area. TPC, total phenolic content; 1, rutin; 2, verbascoside; 3, luteolin-4′-*O*-glucoside; 4, oleuropein; 5, luteolin; 6, quercetin. The same below.

**Table 5:** Quantification of the main phenolic compounds in both aged and young leaves from five major olive cultivars extracted using UAE and WBE methods *.

Varieties	Leaf Age	UAE						
		TPC (mg GAE/g)	1 (%)	2 (%)	3 (%)	4 (%)	5 (%)	6 (%)
Kor	YL	180.72 ± 4.09 a	0.22 ± 0.005 d	0.91 ± 0.03 g	0.46 ± 0.029 b	3.23 ± 0.08 d	0.025 ± 0.0043 c	0.0212 ± 0.0036 abc
	AL	134.40 ± 15.53 cd	0.35 ± 0.014 c	0.30 ± 0.03 i	0.26 ± 0.007 d	0.97 ± 0.01 g	0.035 ± 0.0093 bc	0.012 ± 0.0011 c
Arbe	YL	145.70 ± 3.78 bc	0.34 ± 0.006 c	1.71 ± 0.02 c	0.46 ± 0.009 b	5.57 ± 0.09 c	0.035 ± 0.0053 bc	0.0236 ± 0.0065 abc
	AL	128.39 ± 6.54 cde	0.22 ± 0.014 d	0.57 ± 0.02 h	0.30 ± 0.015 d	1.5 ± 0.04 f	0.032 ± 0.0079 bc	0.015 ± 0.001 bc
Che	YL	171.09 ± 4.66 a	0.07 ± 0.020 e	3.51 ± 0.06 a	0.38 ± 0.004 c	7.46 ± 0.13 a	0.055 ± 0.0132 ab	0.0468 ± 0.0153 a
	AL	108.33 ± 1.64 e	0.05 ± 0.002 e	2.34 ± 0.05 b	0.40 ± 0.007 c	5.39 ± 0.09 c	0.07 ± 0.0067 a	0.043 ± 0.0059 ab
Pic	YL	165.08 ± 6.89 ab	0.05 ± 0.003 e	1.04 ± 0.03 f	0.30 ± 0.009 d	5.59 ± 0.18 c	0.024 ± 0.0034 c	0.0243 ± 0.0067 abc
	AL	121.08 ± 6.14 de	0.05 ± 0.003 e	0.48 ± 0.02 h	0.19 ± 0.007 e	1.37 ± 0.04 f	0.027 ± 0.0062 c	nd
Arbo	YL	165.49 ± 0.53 ab	0.8 ± 0.023 a	1.14 ± 0.02 e	0.47 ± 0.010 b	2.58 ± 0.04 e	0.042 ± 0.0034 bc	0.0155 ± 0.0005 bc
	AL	163.72 ± 6.01 ab	0.47 ± 0.005 b	1.46 ± 0.03 d	0.66 ± 0.018 a	5.97 ± 0.13 b	0.036 ± 0.0066 bc	0.0127 ± 0.0009 c
		**WBE**						
Kor	YL	112.79 ± 1.97 f	0.19 ± 0.022 d	0.58 ± 0.06 g	0.28 ± 0.016 e	1.62 ± 0.38 de	0.017 ± 0.0039 b	nd
	AL	59.01 ± 5.09 h	0.23 ± 0.008 c	0.71 ± 0.03 f	0.31 ± 0.006 d	2.51 ± 0.09 c	0.017 ± 0.0015 b	nd
Arbe	YL	128.41 ± 0.98 e	0.23 ± 0.010 c	1.42 ± 0.06 c	0.36 ± 0.004 b	4.62 ± 0.04 b	0.018 ± 0.0012 b	nd
	AL	72.56 ± 2.12 g	0.14 ± 0.005 e	0.43 ± 0.02 h	0.20 ± 0.019 f	1.32 ± 0.20 e	0.011 ± 0.0003 b	nd
Che	YL	173.29 ± 4.35 b	0.01 ± 0.001 g	2.89 ± 0.03 a	0.28 ± 0.004 e	6.09 ± 0.06 a	0.027 ± 0.0004 b	0.0057 ± 0.0004 b
	AL	158.30 ± 1.73 c	0.05 ± 0.007 f	2.04 ± 0.02 b	0.32 ± 0.004 cd	4.85 ± 0.07 b	0.028 ± 0.0005 b	0.0057 ± 0.0007 b
Pic	YL	163.63 ± 2.65 c	0.06 ± 0.004 f	1.04 ± 0.04 d	0.28 ± 0.011 e	5.65 ± 0.20 a	0.08 ± 0.0102 ab	0.0256 ± 0.0019 a
	AL	142.04 ± 1.72 d	0.05 ± 0.002 f	0.47 ± 0.01 h	0.16 ± 0.008 g	1.38 ± 0.02 e	0.017 ± 0.0008 b	nd
Arbo	YL	217.40 ± 1.26 a	0.54 ± 0.004 a	1.11 ± 0.01 d	0.46 ± 0.007 a	4.6 ± 0.04 b	0.021 ± 0.0012 b	nd
	AL	110.26 ± 4.06 f	0.36 ± 0.005 b	0.89 ± 0.01 e	0.35 ± 0.006 bc	1.94 ± 0.01 d	0.199 ± 0.1115 a	nd

* Results followed by the same lowercase letter are not significantly different according to Duncan’s test (*p* < 0.05); results are expressed as the percentage of dry matter and represent the mean (±SE) of three independent experiments; nd, not detected. Kor, Koroneiki; Arbe, Arbeqina; Che, Chemlal; Pic, Picholine; Arbo, Arbosana. AL, aged leaves; YL, young leaves.

**Table 6:** Independent variables and their levels used for Box–Behnken design.

Independent Variable	Symbols	Factor Level of UAE			Factor Level of WBE		
		−1	0	1	−1	0	1
Extraction time	X_1_	40 min	50 min	60 min	120 s	150 s	180 s
Concentration (%)	X_2_	10	20	30	20	30	40
Liquid–solid ratio (mL/g)	X_3_	100	200	300	100	200	300

## Data Availability

The data that support the findings of this study are available from the corresponding author upon reasonable request.
